# Society Meetings

**Published:** 1899-11

**Authors:** 


### SOCIETY MEETINGS.

The New York State Medical Association held its sixteenth annual meeting at the New York Academy of Medicine, 17 West 43d Street, New York City, Tuesday, Wednesday and Thursday, October 24, 25 and 26, 1899, under the presidency of Dr. Joseph D. Bryant, of New York City. It was a well attended and interesting meeting, and a large number of excellent papers were read.

On Tuesday evening a discussion was had on Medical expert testimony, participated in by the Hon. Willard Bartlett, Justice of the Appellate Division of the Supreme Court; the Hon. Joseph F. Daly, formerly Justice of the Supreme Court; Charlton T. Lewis and William A. Parrington, Esquires, of the New York Bar, and by Dr. Theron A. Wales. On Wednesday afternoon a discussion on typhoid fever was had under the following titles:

1. The advance in our knowledge of typhoid fever, by Herman M. Biggs, of New York City.
2. Bacteriology of typhoid fever, by W. H. Park, of New York City.
3. Clinical diagnosis of typhoid fever, by William Osler, of Baltimore.
4. Statistics of typhoid fever at the Roosevelt Hospital for ten years, by W. H. Thomson, of New York City.
5. Typhoid fever in the Massachusetts General Hospital during the past fifty years, by Reginald Fitz, of Boston, Mass.
6. Some phases of typhoid fever as seen in Bellevue Hospital, by A. A. Smith, of New York City.

7. Typhoid fever in children, by Abraham Jacobi, of New York City.

8. The local—non-surgical—treatment of the intestinal tract in typhoid fever, by De Lancey Rochester, Buffalo.

9. Treatment of perforation of the bowel in typhoid fever, by W. W. Keen, of Philadelphia.

10. Eye complications in typhoid fever, by A. A. Hubbell, of Buffalo.

11. ................., by E. G. Janeway, of New York City.

Wednesday evening, 9.30 to 11.30 o'clock a reception was given at the New York Academy of Medicine to William W. Keen, M.D., LL.D., president of the American Medical Association and to Prof. Reginald H. Fitz, of Boston, and Prof. William Osler, of Baltimore.

---

The Mississippi Valley Medical Association held its twenty-fifth annual meeting at Chicago, October 3-6, 1899. It was considered the most successful meeting in the history of the organisation. The attendance was large and the discussions were spirited. It was determined to publish a volume of Transactions that will be issued promptly. The following were appointed a committee on publication: Drs. Henry E. Tuley, Dudley S. Reynolds and Lewis S. McMurtry. The executive committee has ordered that no volume be sent a member who is in arrears for dues.

The following officers were elected for the ensuing year: President, Harold N. Moyer, Chicago, Ill.; first vice-president, A. H. Cordier, Kansas City, Mo.; second vice-president, S. P. Collings, Hot Springs, Ark.; secretary, Henry E. Tuley, Louisville, Ky.; treasurer, Dudley S. Reynolds, Louisville, Ky. Chairman of committee of arrangements, M. H. Fletcher, Asheville, N. C.

The twenty-sixth annual meeting will be held at Asheville, N. C., October 9-11, 1900.

---

The Southern Surgical and Gynecological Association will hold its twelfth annual meeting at New Orleans, La., Tuesday, Wednesday and Thursday, December 5, 6, and 7, 1899, under the presidency of Dr. Joseph Taber Johnson, of Washington, D. C. The secretary, Dr. William E. B. Davis, of Birmingham, Ala., is preparing an excellent program for the occasion, and the delightful weather prevailing at this season in New Orleans ought to and no doubt will ensure a large attendance.

THE American Public Health Association is holding its twenty-seventh annual meeting just as this JOURNAL is reaching its readers—namely, October 31st to November 3, 1899, at Minneapolis. We have not seen a preliminary announcement of the meeting, but presume it is planned on the usual lines. Dr. Ernest Wende, Health Commissioner of Buffalo, is in attendance, representing the public health interests of this municipality.

---

THE Surgeons' Association of the Western New York & Pennsylvania Railroad, held their seventh annual meeting at Dunkirk, October 11, 1899, and elected the following officers: President, George E. Ellis, of Dunkirk; vice-president, James V. Otto, of Port Allegany, secretary and treasurer, E. M. Dooley, of Buffalo; committee on arrangements, G. E. Ellis, Dunkirk, chairman; J. C. Clark, of Olean, and J. A. Richey, of Oil City, Pa.

A number of Buffalo surgeons read papers on subjects of interest to the Association.

Among the physicians in attendance were: P. L. Van Allen, L. J. McAdam, C. M. Daniels, J. A. Sheridan, Buffalo; William H. Gail, East Aurora; G. E. Ellis, Dunkirk; C. W. Coulter, William Forster, S. W. McDowell, J. P. Strayer, P. M. Speer, J. M. Ward, J. A. Ritchey, F. F. Davis, E. W. Mutheman, I. P. Heindell, Oil City; James V. Otto, Port Allegany, Pa.; Benjamin Chadwick, Smethport, Pa.; J. C. Clark, B. J. Wilmoth, Olean; E. L. Dickey, St. Petersburg, Pa; George H. Kenyon, Providence, R. I.; J. M. Hamilton, Oakmont, Pa.; J. W. Moujar, W. C. Tyler, Rouseville; William Varian, Titusville.

The next annual meeting is to be held in Olean.

---

THE Medical Society of Virginia held its thirtieth annual meeting at the Jefferson Hotel, Richmond, Tuesday, Wednesday and Thursday, October 24, 25 and 26, 1899, under the presidency of Dr. Jacob Michaux, of Richmond. A large number of valuable papers were read and the usual executive business was transacted.

---

THE Medical Association of Central New York held its thirty-second annual meeting at the Yates Hotel, Syracuse, N Y., Tuesday, October 17, 1899, beginning at 10 o'clock in the forenoon. A large attendance of the physicians of Central and Western New York was noted and the meeting was a success. Many interesting papers were presented and read. The discussions were animated and interesting and the best of fellowship prevailed. A dinner was given to the visiting members at the Yates and a complimentary banquet was tendered those of the visitors who sojourned in Syracuse until the late trains.

The officers elected for the year were: President, John Gerin, of Auburn; first vice-president, Lucien Howe, of Buffalo; second vice-president, A. L. Beahan, of Canandaigua; secretary, Charles A. Vander Beek, of Rochester; treasurer, S. L. Elsner, of Rochester. The next meeting will be held at Rochester the third Tuesday in October, 1900. The papers read and presented at the meeting will be published as usual in the BUFFALO MEDICAL JOURNAL.

---

THE Buffalo Academy of Medicine held meetings during the month of October as follows:

*Section on Surgery.*—Tuesday evening, October 3d, program: A report of the operative treatment of seveial cases of frontal and maxillary sinusitis, Frank W. Hinkel; Subphrenic abscess, Prescott LeBreton.

*Section on Medicine.*—Tuesday evening, October 10th, program: Malaria, Irving P. Lyon; discussion by Charles G. Stockton, Marshall Clinton and John D. Howland.

*Section on Pathology.*—Tuesday evening, October 17th, program: A case of poliencephalomyelitis in an adult, presenting the clinical symptoms of Landry's paralysis and terminating fatally in nine days, Dewitt H. Sherman; discussed by James W. Putnam and William C. Krauss. The significance of chronic rhinitis in relation to ocular affections, Edmund E. Blaauw.

*Section on Obstetrics.*—Tuesday evening, October 24th, program: Pus in the pelvic cavity, Dr. Herman E. Hayd.

